# Integrative Approaches to Understand the Mastery in Manipulation of Host Cytokine Networks by Protozoan Parasites with Emphasis on *Plasmodium* and *Leishmania* Species

**DOI:** 10.3389/fimmu.2018.00296

**Published:** 2018-02-23

**Authors:** Anusree Mahanta, Piyali Ganguli, Pankaj Barah, Ram Rup Sarkar, Neelanjana Sarmah, Saurav Phukan, Mayuri Bora, Shashi Baruah

**Affiliations:** ^1^Department of Molecular Biology and Biotechnology, Tezpur University, Tezpur, India; ^2^Institute of Stem Cell Biology and Regenerative Medicine, Bengaluru, India; ^3^Chemical Engineering and Process Development, CSIR- National Chemical Laboratory, Pune, India; ^4^Academy of Scientific and Innovative Research (AcSIR), CSIR-NCL Campus, Pune, India

**Keywords:** cytokine networks, manipulation, *Plasmodium*, *Leishmania*, *inflammation*, signalling hubs, cross regulation, system biology

## Abstract

Diseases by protozoan pathogens pose a significant public health concern, particularly in tropical and subtropical countries, where these are responsible for significant morbidity and mortality. Protozoan pathogens tend to establish chronic infections underscoring their competence at subversion of host immune processes, an important component of disease pathogenesis and of their virulence. Modulation of cytokine and chemokine levels, their crosstalks and downstream signaling pathways, and thereby influencing recruitment and activation of immune cells is crucial to immune evasion and subversion. Many protozoans are now known to secrete effector molecules that actively modulate host immune transcriptome and bring about alterations in host epigenome to alter cytokine levels and signaling. The complexity of multi-dimensional events during interaction of hosts and protozoan parasites ranges from microscopic molecular levels to macroscopic ecological and epidemiological levels that includes disrupting metabolic pathways, cell cycle (*Toxoplasma* and *Theileria* sp.), respiratory burst, and antigen presentation (*Leishmania* spp.) to manipulation of signaling hubs. This requires an integrative systems biology approach to combine the knowledge from all these levels to identify the complex mechanisms of protozoan evolution *via* immune escape during host–parasite coevolution. Considering the diversity of protozoan parasites, in this review, we have focused on *Leishmania* and *Plasmodium* infections. Along with the biological understanding, we further elucidate the current efforts in generating, integrating, and modeling of multi-dimensional data to explain the modulation of cytokine networks by these two protozoan parasites to achieve their persistence in host *via* immune escape during host–parasite coevolution.

## Introduction

Parasitic protozoa are responsible for some of the major diseases of humans affecting several million people each year resulting in significant morbidity and mortality and loss of economic activity. There have been some gains in reducing the incidence of these diseases owing to better intervention strategies, but in absence of effective vaccines, diseases like malaria, leishmaniasis, trypanosomiasis still pose a major public health problem. These protozoans typically establish chronic infections validating their success in evasion and manipulation of host defense and of metabolic processes for their survival, proliferation, and transmission. Many of these pathogenic protozoa have adapted to intracellular habitat as seen in infections by *Plasmodium* spp., *Leishmania* spp., and others. The intracellular niche makes them vulnerable to lysosomal enzymes, reactive oxygen intermediates, and detection by cytosolic sensors of infection, but also offers some protection from adaptive immunity (1). This dynamic host–pathogen interaction, leads to the activation of a series of intracellular and intercellular biochemical signaling processes leading to synthesis of diffusible effector molecules that includes cytokines and reactive oxygen species. “The earliest stages of infection are a parasite’s first opportunity to establish itself within its host and conversely, it is also the host’s chance to mount a rapid and effective response to clear, or at least control the infection” (2). Recent studies demonstrate that pathogens including protozoa modulate the host cell environment by manipulating the host transcriptome by epigenetic modifications besides targeting the major signaling hubs of metabolic, immune, and cell cycle processes to promote their growth, multiplication and survival (3–9). Many protozoans secrete effector molecules that actively modulate host immune transcriptome to alter cytokine levels and signaling either to escape immune processes as in liver stages of *P. falciparum* or to drive their growth as seen in the blood stages of this pathogen.

Considering the diversity of protozoan pathogenesis, this review will focus on manipulation and hijacking of cytokine networks by *Leishmania* and *Plasmodium* spp. for their survival in human host. We will highlight few recently published representative omics and systems biology based studies on *Leishmania* and *Plasmodium* parasites, toward understanding modulation of cytokine and chemokine networks in the host by the parasite to achieve their persistence in host *via* immune escape.

## Cytokines and Cytokine Regulation

Cytokines are small molecules of the immune system, synthesized by various cell types that by virtue of binding to their receptors present on a multitude of cells mediate immune cell activation, differentiation, and cross talk to maintain immune homeostasis (10, 11). Synthesis and regulation of cytokine expression depends on the type of stimulus, cell type, and its state of activation (12–14). Expression of cytokine genes is also regulated by epigenetic modifications that include DNA methylation, histone modifications, and higher order chromatin interactions (15, 16) and posttranscriptional regulation by micro RNA-mediated mechanisms (16–19). Differentiation of immune cells as in T cell subpopulations and macrophage phenotypes is determined and regulated by cytokine environment (4, 16, 20, 21) and epigenetic modifications at cytokine gene loci (22, 23). Cytokine crosstalk between IFNα/β and TNF-α was noted to be at level of chromatin wherein IFNs in addition to regulating interferon signaling genes, also potentiated the TNF genes (4). Similarly, emerging data suggest extensive crosstalk between NLR family proteins of inflammation complex for IL-1β and IL-18 secretion and other cytokines integrated signalosome facilitating integration of diverse pathways for optimal immune response (24). H3K27, methyltransferase enhancer of zeste homolog 1 is reported to promote TLR-triggered inflammatory cytokine production by suppressing the TLR negative regulator toll-interacting protein, thereby contributing to the full activation of the innate immune response against invading pathogens (25).

## Cytokine Signaling Manipulation by Protozoan Pathogens

Intracellular protozoa modulate cytokine gene expression and signaling by some common themes that include targeting of transcription factors (15, 23) phosphorylation status of signaling molecules like STATs, immune check point molecules like CTLA-4 and PD-1 to drive regulatory pathways (26) as well as kinases (5, 6, 27). The pathways usually targeted by pathogens include NF-κB, cell cycle, interferons, MAP Kinase JAK–STAT and pathways mediated by TLR and NLR receptors because of their wide range of functionality and core association with the host genome (28–30).

*Toxoplasma* spp. secrete dense granular protein (GRA) and Rhoptry proteins that activate host kinases and possess kinase activity, respectively, into host cell, which by phosphorylating STAT3 and STAT6, nuclear translocation of NF-κB or activation status of MAPK pathways modulate the levels of IL-4, IL-6, IL-12, and IFN-g (31–35). “*T. gondii* inhibitor of STAT1 transcriptional is another secretory protein that recruits the host nucleosome remodeling and deaceytlase complex to block STAT1-mediated gene transcription” (36). *Trypanasoma cruzi* modulates NF-κB pathway by TLR and NLR mediated signaling for favorable cytokine environment (37–39) However, the protozoa is also reported to manipulate TGF β pathway (40) and also induces the production of IL-10 (40, 41) and arginase for its survival and replication.

## *Plasmodium* and Host Inflammatory Response

Malaria, caused by *Plasmodium* spp. of Apicomplexa phylum, has been the strongest evolutionary selective force in recent human history and has shaped human genome (42) and is one of the major causes of mortality of children below 5 years of age particularly in WHO African region, taking the life of a child every 2 min (43). The life cycle of the parasite is complex and completed in multiple stages in the human and in the mosquito (female *Anopheles* spp.) hosts with stage specific gene and protein signatures (44). Briefly, sporozoites inoculated into human host by bite of infected mosquito travel to liver to mature into merozoites that infect RBCs to continue asexual cycle and also develop into gametocytes which, after fertilization in mosquito gut, develop and mature into sporozoites.

During the liver stages of the parasite, the host immune response tends to be tolerogenic and circumsporozoite protein was seen to inhibit NADPH oxidase and IL-12 and suppressed IL-6 and TNF-α secretion with simultaneous increase of IL-10 levels, allowing parasite to escape detection by immune system (45, 46).

Inflammation is recognized as pivotal feature of immune response to blood stages of *Plasmodium* infection (47). Notably, clinical manifestations of the disease are related to erythrocytic stage of infection. An early and finely balanced inflammatory response with increase in levels of pro-inflammatory IL-12, IFN-γ, TNF-α, IL-1β, and IL-6 and of anti-inflammatory IL-10 and TGF-β is essential for resolution of parasitemia and of disease (48–52). However, pathological activation of exaggerated levels of the very same pro-inflammatory cytokines (cytokine storm) concomitant with lower levels of regulatory mechanisms has been attributed to severe and cerebral malaria syndromes (14, 53–57). A recent study examined the levels of different biomarkers of immune response and found high concentrations of sCDI63 and Fractalkine, which are involved in immune response downregulation and modulation of anti-inflammatory responses in asymptomatic malaria (58). These authors also reported high levels of Neopterin, which is related to increased cell-mediated immune responses and macrophage activation in severe and cerebral malaria patients, indicating an overall sustained state of inflammation supporting the hypothesis of intense and prolonged inflammatory response in severe and in cerebral malaria patients.

The question then arises is that why and how would the parasite drive intense inflammatory response that has the potential to be fatal which could limit parasite transmission and hence not be in interest of the pathogen? The answer appears to lie in (a) enhanced expression of adhesion molecules on endothelial cells by pro-inflammatory cytokines (IFNγ and TNFα) (59) and (b) by requirement for endothelial adhesion mediated by *P. falciparum* membrane protein 1 (PfEMP1) with CD36 and endothelial protein C receptor (EPCR) (60, 61). From the parasite view, endothelial sequestration is essential to escape clearance in spleen and to facilitate *falciparum* merozoite maturation. The highly diverse PfEMP1 proteins encoded by parasite *var* genes contain a Duffy-binding like and cysteine-rich interdomain region (CIDR) domains. Most CIDRα1 domains bind to EPCR and CIDRα2–6 bind CD36 (60, 61). Notably, interaction of EPCR with its ligand the activated protein C (APC) has a role in anti-inflammatory, coagulation homeostasis, and endothelial barrier protection functions (62) and its blockade of these functions by PfEMP1–EPCR interaction that is postulated to contribute to cerebral malaria pathology (59, 61). Interestingly, Smith et al. (61) found increased association of severe malaria with EPCR binding CIDRα1domain containing isolates supporting the contention. Interactions with CD36 are also reported to inhibit IL-12 synthesis and suppressing dendritic cell (DC) maturation and T cell activation.

**Figure 1:**
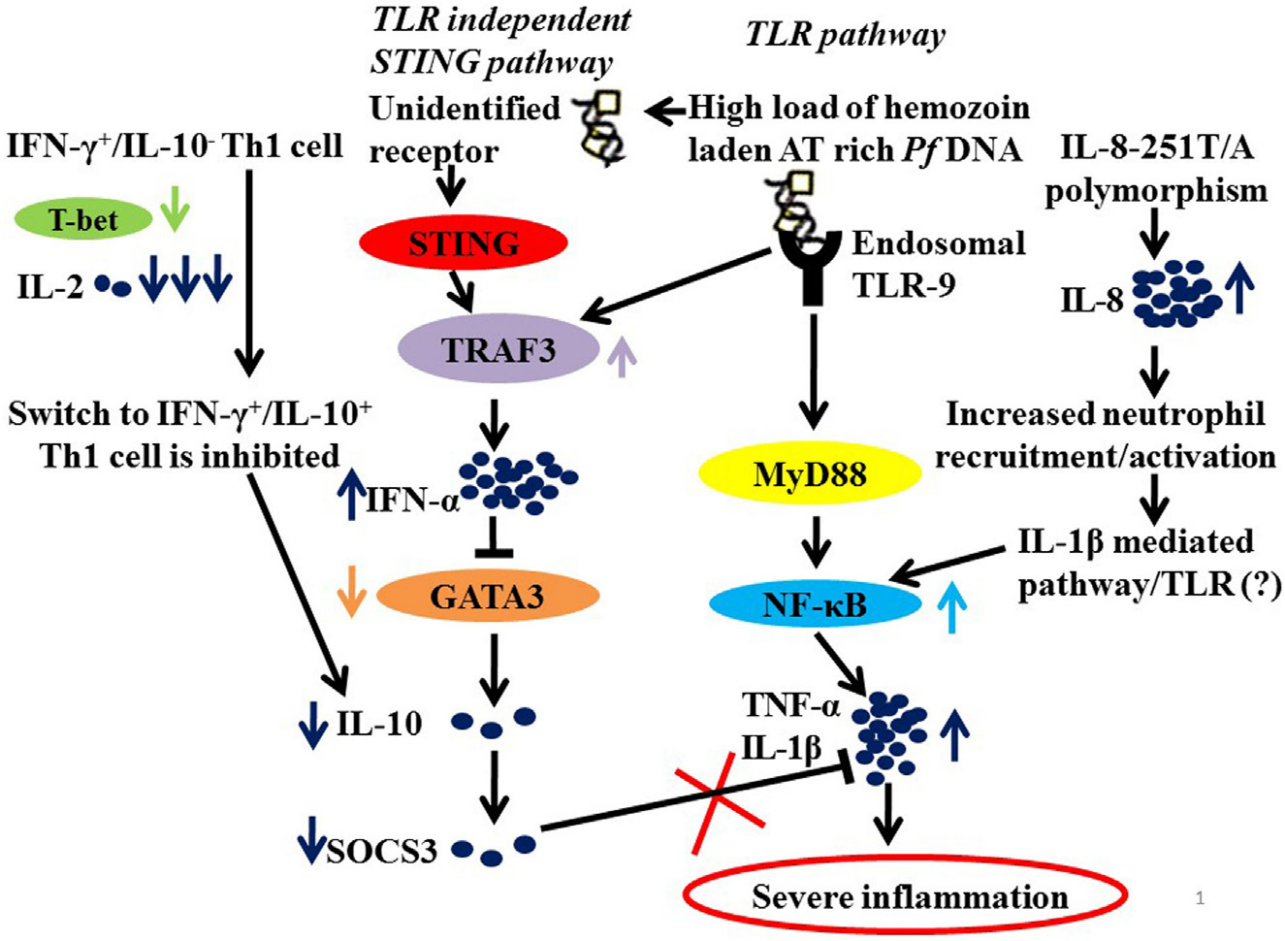
A hypothetical model summarizing the probable mechanisms of severe inflammation in malaria. Parasite molecules like Haemazoin, Pf AT-rich DNA recruited by TLR and TLR independent (STING) pathways (63, 69). High load of Pf AT rich DNA would lead to increased levels of TRAF3 and of IFN-α. And IFN-α, in turn, suppresses GATA3 expression in Th2 cells resulting in low levels of IL-10 and hence down regulated SOCS3 (68). In addition, low levels of IL-2 and T-bet fail to mediate switch from IFN-γ+/IL-10− to IFN-γ+/IL-10+ Th1 cells that requires T-bet and IL-2 levels, also explain low levels of IL-10. Finally, downregulated SOCS3, which is known to mediate the anti-inflammatory functions of IL-10, fails to regulate an exaggerated proinflammatory response. Another contributory role to severe inflammation in malaria is the high prevalence of IL-8-251T/A, which increases IL-8 expression for enhanced recruitment and activation of inflammatory cells neutrophils resulting in increased activation of NF-κB *via* IL-1β-mediated pathway.

It is, therefore, not unimaginable that parasite manipulates NF-κB and Type 1 interferon pathway to drive inflammation. *Plasmodium*-derived PAMPs that include GPI anchors, CpG motifs, AT-rich motifs, and haemazoin are sensed by PRRs of host that include TLRs, NLRs, and AIM2 on cells of monocyte/macrophage lineage and on DCs (61, 63–65). These ligand–receptor interactions initiate MyD88 and STING-IRF3 mediated downstream signaling leading to activation of NF-κB and IRF3 pathways and synthesis of pro-inflammatory cytokines and interferon α/β (55, 65–68). It is the exaggerated activation of these pathways “mediated by IFN-γ pro-inflammatory priming with extreme levels of pro-inflammatory mediators” with concomitant loss of regulatory cytokines that drives malaria pathogenesis (46, 57, 68). It has also been proposed that in addition to driving inflammation, *P. falciparum* by downregulating GATA3 expression suppresses IL-10 and SOCS3 that are necessary to control inflammation, possibly by exploiting the IFNα/β pathway as summarized in Figure 1.

## *Leishmania*: T Cell Differentiation and Cross Regulation of Cytokine Signaling

Leishmaniasis caused by *Leishmania* spp. is a public health problem with 1.3 million reported Leishmaniasis cases worldwide which is intensified by availability of few effective drugs (70) and vaccine (71, 72). Being an intracellular parasite, it needs to overcome host-resistance mechanisms and exploit host environment for survival. From the parasite context, metabolism of *Leishmania* possesses a unique metabolic organization that can re-route metabolites, the uptake of which is constrained in different host environments toward synthesis of specific biomass metabolites; thereby providing novel mechanisms for metabolic adaptations (73, 74). From the host context, the contribution of specific virulence factors in immune suppression or the inability of the host to generate a sufficient immune response against the parasite, which promotes infection. Survival strategy of *Leishmania* is to modulate the signaling pathways of the macrophages after entering the phagolysosome. Depending on the type of infection and the parasite burden, either Th-1 healing or the Th-2 non-healing immune responses are generated, but detailed mechanism is poorly explored. This can be largely understood with respect to the interaction of parasite molecules with the host signaling pathways to suppress host immunity against infection (71).

During invasion, the surface molecules of *Leishmania* interact with the toll-like-receptor proteins present on the macrophages membrane (75). The activation of the TLRs triggers the downstream signaling pathways such as the RAS–RAF-mediated MAPK pathway, canonical and non-canonical NF-κB pathway, JAK–STAT pathway, PI3K–PLC Gamma pathway, and the JNK pathway (76). Subsequently several transcription factors, such as ERK1/2, NF-κB, NFAT, AP1, STAT3, are activated that initiate the synthesis and secretion of several cytokines, growth factors, chemokines and antimicrobicidal molecules which are responsible for the host immune responses during the infection (77).

**Figure 2:**
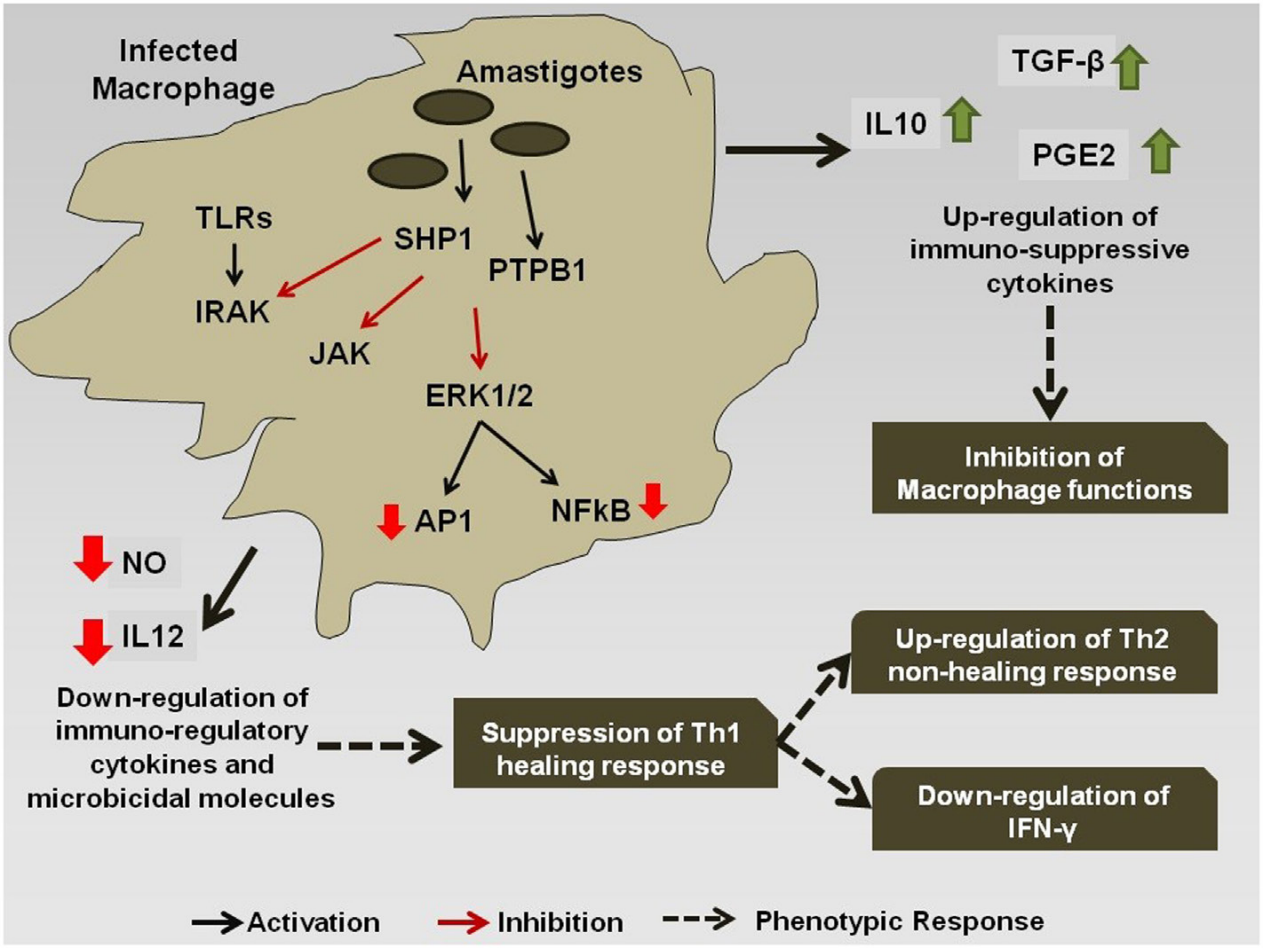
Immuno-modulation by *Leishmania* parasite: *Leishmania* antigens interfere with the signaling cascade of the macrophage and promote the Th-2 non-healing response that helps in the survival of the parasite inside the host.

However, during chronic infection (Figure 2), the antigenic molecules of the *Leishmania* parasite activate the phosphatase proteins in the macrophage, e.g., SHP-1 and PTP1B, which leads to the dephosphorylation and deactivation of selected signaling pathways (78). This leads to downregulation of expression of iNOS and nitric oxide in the infected macrophages, thereby compromising microbicidal functions of the cell and creating an immune-suppressed condition, which is favorable for the continued survival of the pathogen inside APC. Simultaneously, the production of the cytokines, such as IL-12 and TNF-α, gets severely reduced. Such changes in the cytokine expression pattern of the antigen-presenting cells leads to the alteration of the phenotypic responses of the T-cells that now start showing a bias toward the non-healing Th-2 immune response that is characterized by an increased production of IL-4, IL-10, IL-13, and TGF-β cytokines (79), and the suppression of IFN-γ that regulates the healing Th-1 response (71). The transcription factors T-bet and GATA3 play a pivotal role in the regulation of the Th-1/Th-2 ratio during the infection (80). *Leishmania* also inhibits the ability of the host cell for antigen presentation to other immune cells, by repressing the MHC class II gene expression (81) and by modulating the interaction of the co-stimulatory molecules B7-1/CD28 (82) and CD40/CD40L (83).

The difference in the antigenic challenge posed to the host gives rise to differences in expression of the macrophage proteins, as seen in visceral versus the cutaneous infections (84). The difference in macrophage protein expression profile, as exemplified by increased production of COX2 and PGE2 production in case of *L. donovani* infection (as opposed to *L. major*) (85) indicates different *Leishmania* species selectively activate or inhibits different host pathways due to differences in the antigenic challenge. Also, it has been observed in a study that *L. donovani*, which is known to cause visceral leishmaniasis, may in rare cases give rise to cutaneous leishmaniasis (86). This behavior of *L. donovani* infection may be attributed to host’s resistance to the disease which restricts the spread of the infection to the visceral organs and keeps it localized to cutaneous regions (86).

The CD4+ CD25+ regulatory T cells also play a major role in regulating the persistence of the parasite *L. major* inside the host. Inhibition of the T-reg promoting cytokines such as IL-10 leads to the clearance of the pathogen from the host (87). However, during Leishmaniasis the low production of the IFN-γ and IL-12 cytokines leads to the increased proliferation of the T-reg cells that leads to the re-activation of the *Leishmania* parasites inside the host (87).

## Systems Biology Based Integrative Approaches for Understanding the Host–Parasite Interaction and Co-Evolutionary Patterns in Protozoan Diseases

During the interaction of hosts and protozoan parasites, both employ mutual selective pressures on each other, which may facilitate rapid reciprocal adaptation. Different stages of the parasite life cycle introduce another layer of complexity (88). Significant amount of molecular, omics, clinical, epidemiological as well as ecological data has been generated at *in vitro* and *in vivo* levels using various pathogens and respective diseases. Integrative analysis of such discretely generated and located data from the host and protozoan parasite variants, in laboratory as well as natural populations is the most essential necessity to identify the complex mechanisms of protozoan evolution *via* immune escape during host–parasite coevolution. Public resources such as EuPathDB (89), Pathogen–Host Interactions (90), ProtozoaDB (91), together with protozoan species-specific databases are tremendously useful to collect useful information for initiating systems based integrative analysis. The key steps in such integrative approach involves data generation/data collection, data organization, data integration, integrative network construction, network analyses, and finally computer-based mathematical simulation and predictive modeling (92). As an example, using a reconstructed genome scale metabolic model of *Leishmania infantum* adaptations, (73) have identified the robustness of the parasite metabolic network against accidental errors and demonstrated the wide array of choices for the parasite to achieve optimal survival (73).

Recent advancement in RNA-Seq based techniques has facilitated the simultaneous sequencing of both host and parasite (including non-model parasites) transcriptomes (93). In a first of its kind RNA-seq experiment in control human neutrophils during priming with pro-inflammatory cytokines (TNF-α and GM-CSF), Wright et al. have shown the rapid expression of a common set of transcripts for cytokines, chemokines, and cell surface receptors (CXCL1, CXCL2, IL1A, IL1B, IL1RA, ICAM1) (94). They have demonstrated the utility of this approach to define functional changes in neutrophils following cytokine exposure. During a mega scale analysis of 116 malaria patients and infecting *P. falciparum* parasite, Yamagishi et al. have identified variable behaviors of the field malaria parasites, which were far more complex than those observed under laboratory conditions (95). Pittman et al. have generated a large scale *T. gondii*–host interactome, using dual transcriptional profiling of mice and parasite during acute and chronic infection (96) to demonstrate the influence of parasite development on host gene transcription as well as the epigenetic influence of the host environment on parasite gene transcription. Various systems-wide studies on malaria parasites have reported posttranscriptional (97) and translational (98) control at various points of the parasite lifecycle. One of such controlling mechanism is translational delay, by which protein expression in parasite is actively suspended for expressed mRNA transcripts. It was shown in *P. falciparum* that by suppressing more than 30% of its genes, the parasite rapidly adapts to new environments within the host by remaining undetected to the host immune system and undergo developmental switching in order to survive (99).

## Conclusion and Future Perspectives

There is large apparent heterogeneity in offense strategies employed by the protozoan pathogen in human infections. In contrast to this, there appears to be a broad consensus on the major signaling hubs manipulated by the pathogens. It would be worthwhile to dissect the host–pathogen interactions at cellular, molecular, and systems level to discriminate between infections that are virulent with potential for fatal outcomes from asymptomatic or uncomplicated infections with limited morbidity. It may be hypothesized that immuno regulatory mechanisms that confer disease tolerance are distinct from immune and metabolic responses to severe diseases and demand to be determined by large global studies employing different protozoan pathogen systems. However, despite the availability of huge amount of multi-dimensional data in host–protozoan interaction, functional characterization, and annotation of parasite genomes is severely limited by lack of both genetic tools and resources in protozoa. Given the size, heterogeneity and complexity of the host–parasite interaction data, development of new computational tools and user-friendly methods for integrating heterogeneous “Big Data” will facilitate to fill up the missing links. This will be beneficial for better understanding of the evolutionary arm race between the host and the parasite, and finally for the efficient management and control of the protozoan diseases in humans.

## Author Contributions

SB: manuscript design and contributed the introduction and sections on cytokines, malaria and *Toxoplasma* and future perspectives, PB: contributed the section on systems biology-based integrative approaches to understand host–parasite interaction and future perspectives, RRS: contributed in the sections on Leishmaniasis and *Trypanasoma*, AM: contributed to the section on malaria, NS: contributed to the section on cytokines and cytokine regulation, SP: contributed to the section on *Toxoplasma*, MB: contributed to the section on *Toxoplasma*, and PG: contributed in the sections on Leishmaniasis and *Trypanasoma*. SP and MB: contributed to section on Cytokine Signalling Manipulation by Protozoan parasites.

## Conflict of Interest Statement

The authors declare that the research was conducted in the absence of any commercial or financial relationships that could be construed as a potential conflict of interest. The reviewer DN and handling editor declared their shared affiliation.
